# Alkali Cations Promote CO_2_ Electroreduction on Cu(100) Surfaces under Acidic Conditions by Suppressing Surface Hydrogen Passivation: A Multiscale Modeling Perspective

**DOI:** 10.1021/jacs.6c05861

**Published:** 2026-06-25

**Authors:** Ke Ye, Qin-Kun Li, Min Hu, Guozhen Zhang, Mårten S. G. Ahlquist

**Affiliations:** † Department of Theoretical Chemistry and Biology, 7655 KTH Royal Institute of Technology, 106 91 Stockholm, Sweden; ‡ Walker Department of Mechanical Engineering, 12330 The University of Texas at Austin, Austin, Texas 78712, United States; § School of Arts and Sciences, 672513 Fuyao University of Science and Technology, Fuzhou 350109, China; ∥ Hefei National Research Center for Physical Sciences at the Microscale, School of Chemistry and Materials Science, 12652 University of Science and Technology of China, Hefei 230026, China

## Abstract

The promotional effect of cations on the CO_2_ reduction reaction (CO_2_RR) on Cu is well-established experimentally, yet the underlying mechanism remains debated. This is further complicated by an underexplored factor: in acidic solution, the Cu surface may be covered by a H adlayer rather than being pristine. We employ a multiscale modeling approach to investigate how the H adlayer and electric double layer environment affect CO_2_RR activity on Cu(100). GC-DFT calculations demonstrate that the H adlayer on Cu lowers the d-band center of Cu, attenuating CO_2_RR activity and correctly identifying CO_2_ chemisorption as the rate-limiting step. This H-passivated surface provides a novel lens through which to view the cation effects. Molecular dynamics simulations reveal that the direct Cs^+^ stabilization of intermediates is a minor effect. More importantly, Cs^+^ thermodynamically favors the transition to lower H coverage and suppresses H_3_O^+^ adsorption on the Cu surface. We thus propose a dual-role mechanism: alkali cations promote CO_2_RR also by acting as depassivants that reduce H coverage, restoring the catalytic activity of the Cu surface for the CO_2_RR.

## Graphical abstract

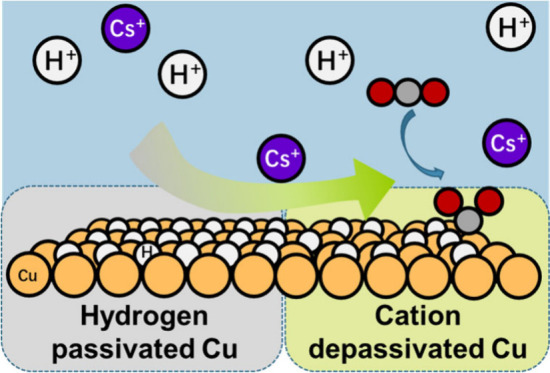

Developing efficient and selective electrocatalysts for the carbon dioxide reduction reaction (CO_2_RR) is one of the major research focuses for addressing energy and environmental challenges.^1–7^ Among candidate materials, copper (Cu) is unique in its ability to catalyze the formation of multicarbon products such as ethylene and ethanol under ambient conditions.^8–11^ Especially acidic CO_2_RR eliminates the carbonate formation bottleneck of alkaline systems and capitalizes on a mature proton exchange membrane, marking substantial progress toward practical electrochemical CO_2_ conversion.^12–15^ Beyond active site engineering, the electric double layer (EDL), including cation^16–28^ and anion^28–31^ species, has proven instrumental in tuning CO_2_RR selectivity and activity.^32^ Among these, cations are especially potent regulators for CO_2_RR.^18,19,33–39^ Their accumulation in the EDL suppresses the hydrogen evolution reaction, enabling CO_2_ reduction even in acidic electrolytes.^36^ Notably, Koper and co-workers found that Cu, Ag, and Au surfaces exhibit no CO_2_-to-CO activity in acidic electrolytes without Cs^+^,^40^ highlighting the indispensable role of cations.

Several mechanisms have been proposed for cation-enhanced CO_2_RR, including electrostatic stabilization of intermediates (*COO^–^, *OCCO),^33,40–42^ electric field (EF) effect,^24,26,43,44^ and regulation of interfacial solvation structure^45^ and local pH.^46–48^ These mechanisms highlight how cations modulate intermediate binding and the local environment, offering design principles for the CO_2_RR catalyst development. Most theoretical studies of cation effects assume clean Cu surfaces, neglecting adsorbate layers under the electrochemical conditions. However, accumulating evidence shows that metal surfaces under operating conditions are typically covered with adsorbates that profoundly influence catalytic performance.^49,50^ Given the dynamic nature of the Cu surface under electrochemical conditions,^29,50–52^ a fundamental question is what the true surface state is and how it impacts the cation effect during electrochemical conversion. Although operando observation of the H adlayer under CO_2_RR conditions remains challenging, computational studies indicate that H adsorption on Cu is thermodynamically favored in acidic media at negative potentials.^53–55^ This raises a critical mechanistic question: how would the H adlayer influence the catalytic cycle and the role of the alkali cations? Answering this could offer new insights into improving electrocatalysts.

In this work, we employ grand-canonical density functional theory (GC-DFT) calculations and classical molecular dynamics (MD) simulations to investigate the coupled effects of the H adlayer and alkali cations on CO_2_RR over Cu(100). By integrating these computational approaches, we elucidate how cations within the EDL enhance the CO_2_RR on the H-covered Cu(100) surface. The results reveal that the H adlayer passivates Cu(100) and suppresses its CO_2_RR activity, while cations not only promote CO_2_ adsorption through electrostatic interactions but also reduce H coverage on Cu, thereby depassivating the Cu surface and restoring its intrinsic CO_2_RR activity. These findings provide mechanistic insights for designing CO_2_RR electrocatalysts.

As shown in Figure 1, GC-DFT calculations at −0.83 V_RHE_ reveal that CO_2_ preferentially adsorbs at the hollow site of the Cu(100) (−0.57 eV) over the top (−0.50 eV) and bridge (−0.30 eV) sites. The adsorbed CO_2_ adopts a bent, negatively charged configuration, indicating thermodynamically favorable activation on Cu(100).

**Figure 1:**
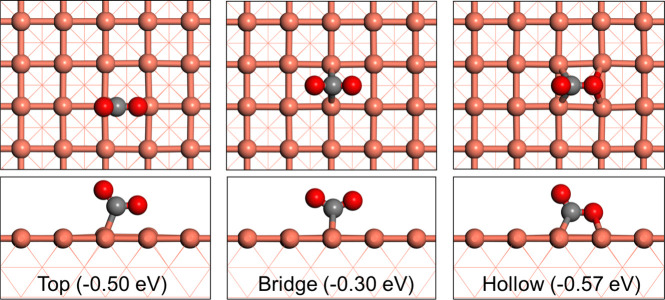
Top and side views of CO_2_ adsorption configurations on Cu(100) at −0.83 V_RHE_: top, bridge, and hollow sites (left to right). Values in parentheses are CO_2_ adsorption free energies.

Figure 2 illustrates the energy profile for CO_2_ to *CO conversion at −0.83 V_RHE_ in pure water, with *COO and *COOH identified as key intermediates. The overall pathway is thermodynamically downhill, indicating spontaneous *CO formation. As shown in Figure 2b, the CO_2_ adsorption barrier is only 0.09 eV on pristine Cu(100), suggesting facile chemisorption. This contrasts with experimental observations identifying CO_2_ adsorption as the rate-determining step.^40^ For the two subsequent protonation steps (*COO + H_3_O^+^ → *COOH + H_2_O and COOH + H_3_O^+^ → CO + H_2_O), the energy barriers (Figure 2c,d) are below 0.16 eV, readily surmountable at room temperature. These results suggest that the pristine Cu(100) is highly reactive for CO_2_ to *CO conversion in acidic media, contradicting experimental observations that Cu is inactive without Cs^+^.^40^ This discrepancy suggests that assuming an idealized clean Cu surface fails to capture the true CO_2_RR under acidic electrochemical conditions.

**Figure 2:**
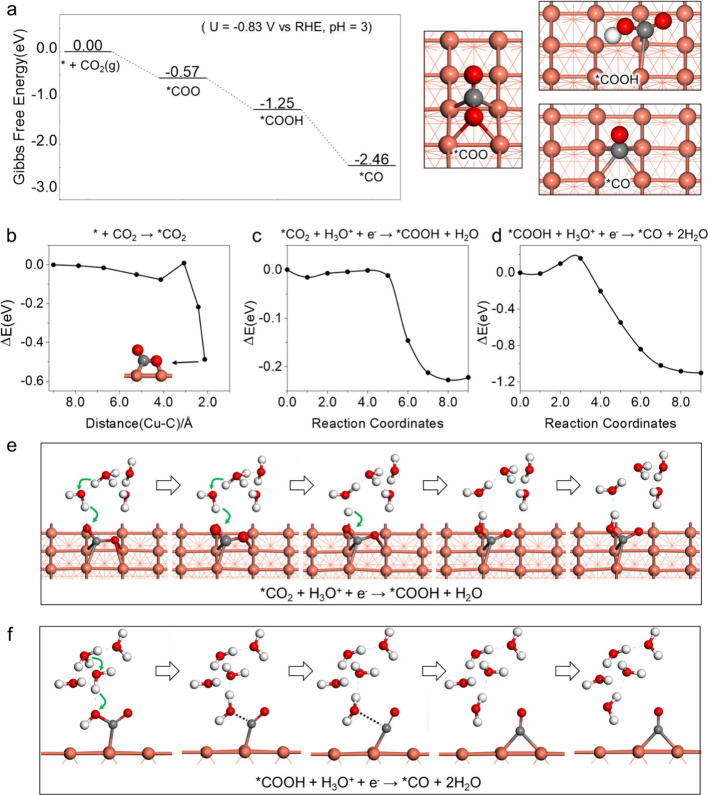
Energy profiles and structural transformations for CO_2_ to *CO conversion on pristine Cu(100) under acidic conditions. (a) Energy profile with representative structures of key intermediates (*COO, *COOH, and *CO). (b) Energy profile for CO_2_ chemisorption at the hollow site, with the Cu–C distance as the reaction coordinate. (c, d) Energy profiles for the first (c) and second (d) protonation steps. (e, f) Corresponding intermediate structures for the first and second protonation steps.

Under acidic conditions, previous computational studies predict that H coverage on Cu exceeds 1.0 ML at working potentials.^53,54^ As shown in Figure 3a, we investigated the effect of H coverage on the catalytic activity of Cu(100) at six coverage levels (0, 0.2, 0.4, 0.6, 0.8, and 1.0 ML). GC-DFT calculations show that H preferentially occupies hollow sites, the same sites favored by CO_2_ adsorption. Increasing H coverage progressively shifts Cu d-band center downward (Figure 3b), suggesting that H adsorption diminishes the CO_2_RR activity of Cu(100) according to d-band center theory. At 0.9 ML H coverage (Figure 3c), CO_2_ adsorbs most stably near unoccupied hollow sites with an adsorption energy of 0.05 eV. Cu–C bond-length scanning reveals a slightly higher adsorption barrier compared to bare Cu(100), indicating a reduction in CO_2_RR activity at 0.9 ML H coverage. However, at 1.0 ML H coverage (Figure 3d), geometry optimization yields no stable CO_2_ adsorption configuration, and Cu–C bond-length scanning reveals that the CO_2_ adsorption barrier rises above 0.90 eV. To understand the substantial barrier difference between 0.90 and 1.0 ML, we tested CO_2_ adsorption at H-occupied hollow sites on the 0.90 ML surface. The results are similar to those at 1.0 ML: geometry optimization yields no stable CO_2_ adsorption configuration, and the adsorption barrier also exceeds 0.90 eV (Figure S3), indicating that the substantial energy difference primarily originates from the H atom at the hollow site directly beneath the adsorbed CO_2_, which strongly suppresses CO_2_ adsorption. These results are consistent with experimental observations that CO_2_ adsorption is the rate-determining step for CO_2_RR to CO on Cu under acidic conditions.^40^ Furthermore, GC-DFT calculations show that increasing H coverage on the stripelike surface progressively shifts the d-band center downward, suggesting that H-induced passivation of CO_2_RR activity extends to the reconstructed surface as well (Figure S4). These GC-DFT calculations reveal that under acidic conditions, Cu(100) is likely covered by H at coverages potentially exceeding 1.0 ML, consistent with previous studies.^54^ H adsorption at hollow sites strongly suppresses CO_2_ adsorption, rendering the Cu surface catalytically inactive for CO_2_RR. Therefore, the H-adsorption/desorption equilibrium serves as a critical determinant of CO_2_RR activity, any shifts induced by environmental variables may significantly alter the catalytic outcome.

**Figure 3:**
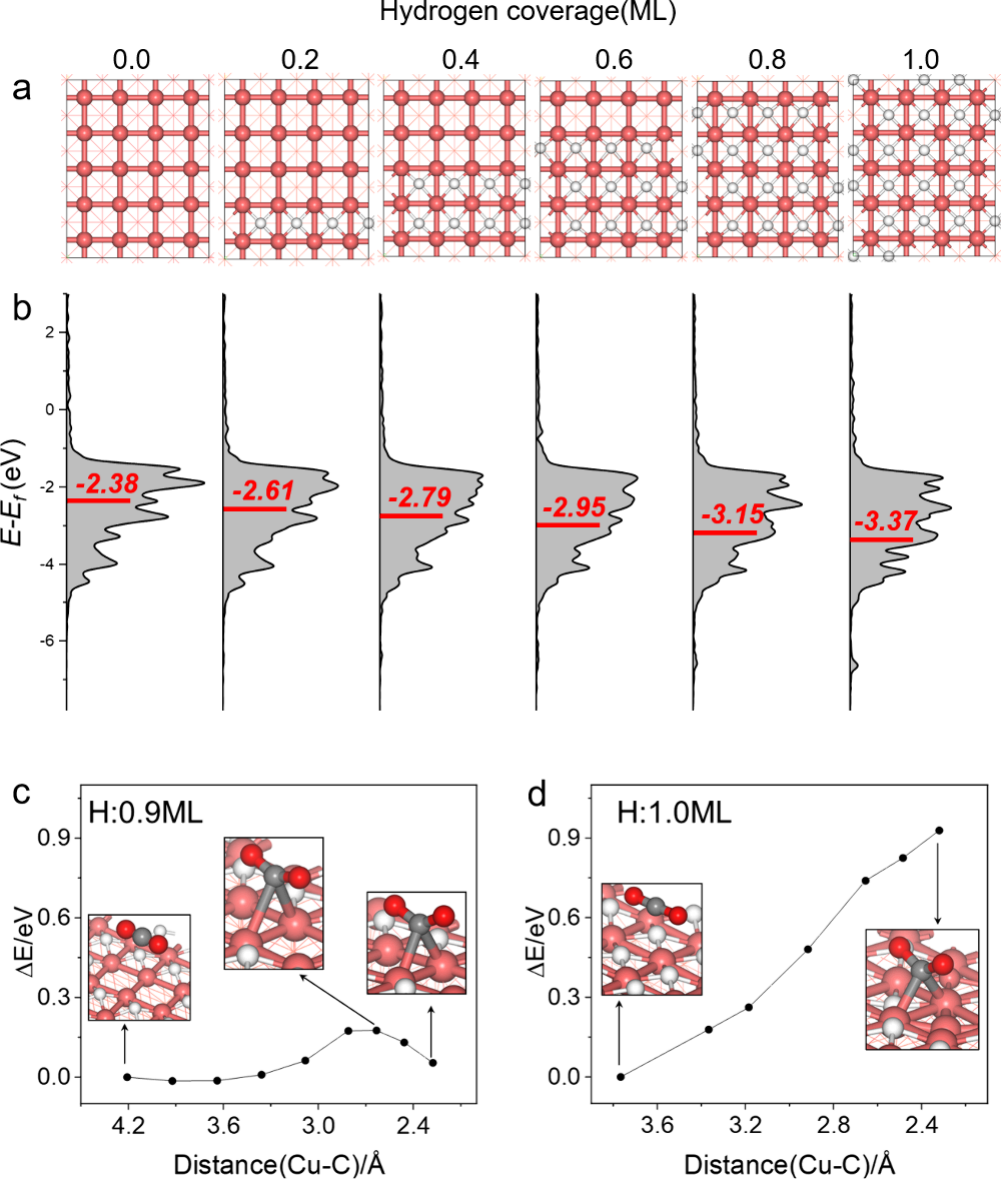
(a) Structure of Cu(100) with varying H coverages. (b) Projected density of states (DOS) of Cu with varying H coverages and the corresponding d-band center positions (numbers in red). (c, d) Energy profiles for CO_2_ adsorbed on Cu(100) at H coverages of 0.9 and 1.0 ML.

Since the above GC-DFT simulations were performed in pure water and the EDL is known to significantly influence CO_2_RR on Cu, we employed classical MD simulations to investigate the effect of the EDL on CO_2_ adsorption on Cu(100) at 1.0 ML H coverage. As illustrated in Figure 4a, the EDL was modeled within a 61 Å × 64 Å × 124 Å simulation cell containing explicit water molecules, Cs^+^, and HSO_4_^–^ ions. MD simulations were performed under a constant EF of −0.60 V/nm to mimic the operational electrode potential. To quantify the effect of the EDL on the free-energy landscape of CO_2_ adsorption, we performed free energy perturbation (FEP) simulations (Figure 4b). This approach allows individual EDL components to be selectively switched on or off, enabling systematic deconvolution of their contributions to the free energy landscape. Four scenarios were modeled to isolate the driving forces behind CO_2_ adsorption: (1) water only (baseline), (2) water with Cs^+^, (3) water with EF, and (4) the complete EDL (water + Cs^+^ + EF). As depicted in Figure 4c, the complete EDL (water + Cs^+^ + EF) significantly promotes CO_2_ adsorption, lowering the adsorption free energy by −0.44 eV (green bar). Decomposition of individual contributions reveals that the EF is the dominant factor (−0.42 eV orange bar), while Cs^+^ provides only a marginal stabilizing effect (−0.08 eV blue bar). The marginal stabilization by Cs^+^ suggests that simple electrostatic stabilization between Cs^+^ and *COO^–^ is insufficient to explain the complete absence of *CO production in Cs^+^ free acidic electrolytes.^40^ This implies that the promotional role of Cs^+^ may extend beyond direct stabilization of *COO^–^. Given that CO_2_ adsorption weakens sharply above 1.0 ML H coverage, we hypothesized that Cs^+^ might promote CO_2_RR by shifting the H adsorption/desorption equilibrium toward lower coverage. MD simulations were then performed to investigate the effect of Cs^+^ on H coverage.

**Figure 4:**
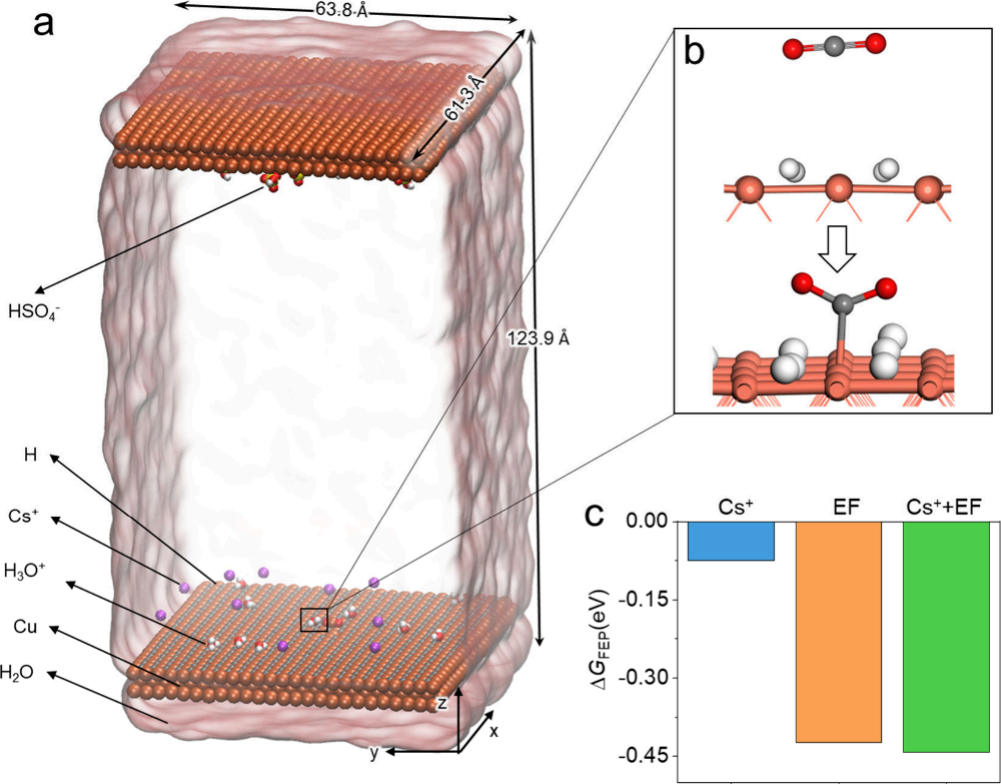
(a) MD simulation box used for FEP calculations. (b) CO_2_ adsorption process on a 1.0 ML H-covered Cu(100) surface. (c) FEP results on the effects of EDL components on the CO_2_ adsorption process. Δ*G*_FEP_ of CO_2_ adsorption in the pure water environment is used as the baseline.

Figure 5a shows the distribution of Cs^+^ above the Cu(100) as a function of H coverage. As the H coverage increases, the Cs^+^ distribution peak weakens and shifts progressively away from the surface, indicating that the H adlayer substantially weakens the interaction between Cs^+^ and Cu surface. To assess the effect of Cs^+^ on H coverage, we performed FEP simulations of the H coverage transition from 1.0 to 0.8 ML on Cu(100), with and without Cs^+^ in the EDL (Figure 5b). FEP results show that the presence of Cs^+^ in the EDL lowers the free energy change for this process by 4.82 eV relative to the Cs^+^-free condition (approximately 1.01 kcal/mol per H atom removed). This indicates that Cs^+^ thermodynamically favors the reduction of H coverage. Furthermore, we performed potential of mean force (PMF) simulations for one H_3_O^+^ migration from the bulk solution to the Cu surface with and without Cs^+^ in EDL (Figure 5c). The results show that Cs^+^ raises the PMF for H_3_O^+^ migration to the Cu surface by ∼7 kcal/mol, suppressing H_3_O^+^ adsorption on the Cu surface, consistent with previous experimental observations that alkali metal cations suppress hydronium reduction.^36,56^ Taken together, these MD simulations demonstrate that Cs^+^ reduces H coverage on Cu(100) through two mechanisms: (1) thermodynamically favoring the transition from high to low H coverage and (2) suppressing H_3_O^+^ migration from bulk solution to Cu(100). These findings suggest that under acidic conditions, Cs^+^ also acts as a depassivant by reducing H coverage, thereby indirectly facilitating CO_2_ adsorption.

**Figure 5:**
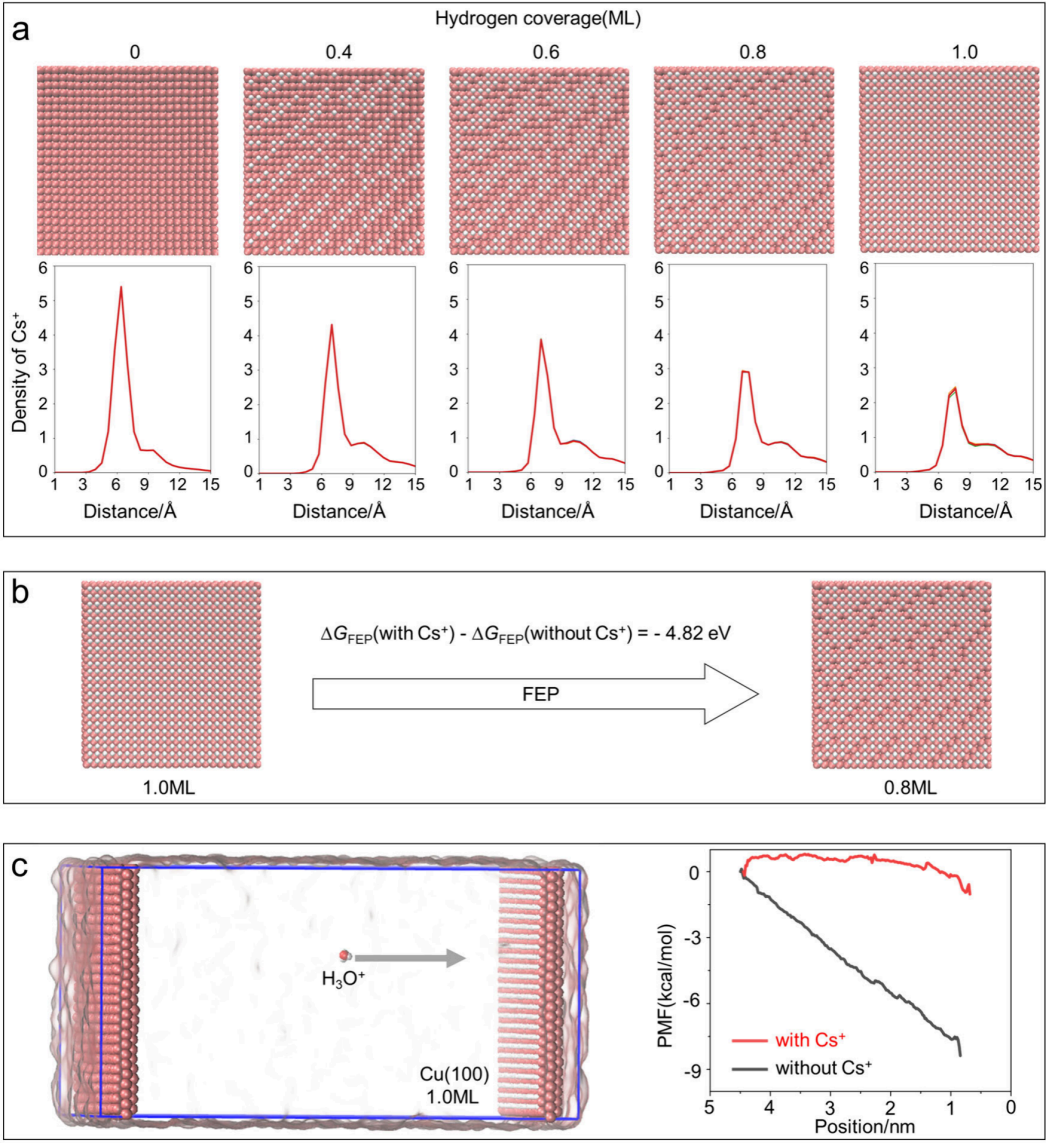
(a) Cs^+^ distribution above Cu surfaces with varying H coverages. As the H coverage increases, the intensity of the Cs^+^ distribution peak decreases, and the peak position shifts progressively farther from the Cu surface. (b) FEP simulations of the free energy change for H coverage transition from 1.0 to 0.8 ML with and without Cs^+^ in the EDL. (c) PMF simulations of H_3_O^+^ migration from bulk solution to the Cu surface with and without Cs^+^.

In this work, we integrate GC-DFT and MD simulations to elucidate the effects of the H adlayer and Cs^+^ in the EDL on CO_2_RR on Cu(100) under acidic conditions. We find that the H adlayer passivates the Cu surface by inhibiting CO_2_ adsorption, making it the rate-determining step. The EDL substantially promotes CO_2_ adsorption, primarily through the interfacial EF, while direct electrostatic stabilization by Cs^+^ plays only a minor role. Notably, Cs^+^ reduces H coverage on Cu(100) through two mechanisms: (1) thermodynamically favoring the transition from high to low H coverage and (2) suppressing H_3_O^+^ migration from bulk solution to the Cu surface. This depassivation effect is likely a key factor underlying the Cs^+^-induced enhancement of the CO_2_RR on Cu under acidic conditions. While the classical MD approach employed here captures long-range EDL interactions at a low computational cost, the neglect of polarization effects remains a limitation. Machine learning force fields (MLFFs) offer a promising alternative with DFT-level accuracy, although accurately capturing long-range EDL interactions remains a challenge for most current implementations. As MLFF methods continue to mature, they may provide a more accurate and efficient approach to EDL simulations.

## Supplementary Material



## Notes

All details of the computational study, including force field parametrization and molecular dynamics simulations; topology file including all parameters used for free energy perturbation; Cartesian coordinates of optimized structures from GC-DFT calculations (PDF)
