# Supplementary material for: Alkali Cations Promote CO2 Electroreduction on Cu(100) Surfaces under Acidic Conditions by Suppressing Surface Hydrogen Passivation: A Multiscale Modeling Perspective
Source: J Am Chem Soc. 2026 Jun 25;148(26):27035–41. doi: 10.1021/jacs.6c05861 (PMC13352604; doi:10.1021/jacs.6c05861)
Supplement: Supplementary file 1 [file ja6c05861_si_001.pdf]

Supporting Information

Alkali Cations Promote CO_2 Electroreduction on Cu(100) Surfaces under Acidic Conditions by Suppressing Surface Hydrogen Passivation: A Multiscale Modeling Perspective

Ke Ye[1], Qin-Kun Li[2], Min Hu[3], Guozhen Zhang[4]*, Mårten S. G. Ahlquist[1]*

[1]Department of Theoretical Chemistry and Biology, KTH Royal Institute of Technology, 10691Stockholm, Sweden
[2]Walker Department of Mechanical Engineering, The University of Texas at Austin, Austin, TX 78712
[3]School of Arts and Sciences, Fuyao University of Science and Technology, Fuzhou, China
[4]Hefei National Research Center for Physical Sciences at the Microscale, School of Chemistry and Materials Science, University of Science and Technology of China, Hefei 230026, China.

Corresponding Authors

Mårten S. G. Ahlquist Email: ahlqui@kth.se

Guozhen Zhang Email: guozhen@ustc.edu.cn

1 Computational Details

1.1 Grand Canonical Ensemble DFT(GC-DFT)

In this study, all GC-DFT calculations were performed with the Vienna Ab Initio Simulation Package (VASP)[1-2], employing the spin-polarized plane-wave density functional theory (DFT) framework based on the generalized gradient approximation(GGA) and the Perdew−Burke−Ernzerhof (PBE) exchange-correlation functional[1, 3-4]. The projector-augmented-wave pseudopotentials were utilized, and the plane-wave basis was truncated at cutoff energy of 450 eV[2, 5]. Long-range van der Waals interactions were addressed employing Grimme's DFT-D3 scheme[6]. The convergence thresholds for the electronic and ionic optimizations were set to 1×10^{-5} eV for the total energy and 0.02 eV/Å for the forces, respectively. The Brillouin zone was sampled with a 3×3×1 Monkhorst–Pack k-point mesh for Cu(100) surface spuercells, and a vacuum spacing of 18 Å was introduced along the surface normal to eliminate the spurious interlayer interactions. Implicit solvation effects were included via the VASP-sol[7], using a dielectric constant of 78.4 to represent water and a debye screening length of 3 Å/sqrt (ionic concentration) corresponding to a 1 M electrolyte[8]. To capture electrochemical behavior under realistic conditions, CO_2 reduction reactions(CO_2RR) energetics were further evaluated under a constant potential framework rather than the constant charge scheme. The GC-DFT approach, implemented following the Duan and Xiao method[9-10], was adopted, where the number of electrons in the system is iteratively updates the total number of electrons in the system until the desired voltage is matched. The obtained grand potential of each state is a Legendre transform of the Gibbs free energy. In addition, we calculate the energy profile of the CO_2 reduction at the constant potential condition, which is closer to the real electrochemical condition than the constant charge condition.

1.1.1 Free Energy Calculation

A PBE-energy correction for small molecules, as proposed by Nørskov et al.[11],

was applied in this study.

The Gibbs free energies of each gas phase molecule was calculated using the following formula:

$$G = E_{DFT} + ZPE - TS + \int C_p \, dT$$

where E_{DFT} is the electronic energy obtained from DFT calculations, ZPE is zero-point energy, T is the temperature (T=298.15K in this work). The entropy S and C_p are found from the NIST Standard Reference Database[10]. After applying a PBE-energy correction for all small molecules developed by Nørskov group,[11] the resulting gas-phase free energy values of all small molecules are summarized in Table S1.

1.1.2 Grand Potential Calculation

When using the constant potential method within the framework of GC-DFT, the Gibbs free energy change is evaluated according to the following formula[12]:

$$\Delta\Omega(E) = \Delta U\{q(E)\} + \Delta ZPE - T\Delta S - \Delta N_{H^+}\{\tfrac{1}{2}\mu_{H_2} - e(E - E_{SHE})\} - \Delta qE$$

Here, $U\{q(E)\}$ represents the DFT-calculated internal energy for a given total charge q, which is a function of E within the grand canonical ensemble. ZPE is the zero-point energy, T is the temperature (T=298 K), and S is entropy, The ZPE and TS terms are computed through vibrational analysis. ΔN_{H^+} is the number of added protons in that reaction, μ_{H_2} is the chemical potential of H_2 gas, E is the absolute potential, and E_{SHE} is the electrode potential of the standard hydrogen electrode (SHE). In GC-DFT calculations, the number of electrons (which determines the value of q) is iteratively updated to tune the Fermi level of the surface until the difference between it and the Fermi level of the reference electrode (typically SHE) matches the desired voltage[13] and the geometry of the system is relaxed in the updated potential energy surface6 until the criteria of convergence are met.

1.2 Classical Molecular Dynamics

To investigate the effects of the EDL environment (including cations and the interfacial electric field) on CO_2 adsorption, we employed classical MD simulations. This is because the EDL typically extends over length scales of >10 nm with some dynamic processes occurring on very long timescales. For example, the mean residence time of water in the hydration shell of Li^+ can be as high as 400 ps.[14] GC-DFT-based MD is generally limited to small system sizes (<200 atoms) and short timescales (<20 ps), making adequate sampling of the EDL interface extremely challenging. Classical MD simulations, by contrast, enable modeling of the EDL at length scales >10 nm and timescales >10 ns, ensuring far more thorough sampling. Since both the electric field and cations are components of the EDL, treating them within the same framework facilitates a consistent analysis and direct comparison of their respective contributions. We acknowledge that classical MD is less accurate than GC-DFT-MD simulations, with the primary source of error in our system being the neglect of the polarization effect. Nevertheless, it offers a significant advantage in sampling that is otherwise difficult to achieve. We have previously applied this approach to successfully reproduce the experimentally observed cation activity trend in EDL-promoted CO_2RR ($Li^+ < Na^+ < K^+ < Cs^+$)[15], and the support-mediated modulation of reaction selectivity at the EDL interface[16]. This hierarchical modeling strategy has been widely adopted for investigating electrochemical reactions and has yielded significant insights.[17]

All Molecular Dynamics (MD) simulations were performed using GROMACS 2019.3 package[18]. Energy minimization was first performed on the box containing 1.0ML H coveraged Cu(100) surface immersed in water or electrolyte. Then a 100 ps simulation with timestep 1 fs was performed under NVT and thereafter under NPT to ensure its stability. To avoid self-interactions under periodic boundary conditions, a sufficiently large box of $63.8 \times 61.3 \times 123.9$ Å^3 was used for all simulations.

1.2.1 Forcefield Parameterisation

The Lennard–Jones (L-J) parameters for Cu atoms were adopted from the work

of Heinz et al.[19], which provides accurate 12–6 and 9–6 LJ potentials for face-centered cubic (fcc) metal surfaces and interfaces. These parameters have been extensively validated to reproduce densities, surface tensions, interface properties with water and (bio)organic molecules, as well as mechanical properties in quantitative (<0.1%) to good qualitative (25%) agreement with experiment under ambient conditions. In our simulations, the Cu–Cu interactions were described using the 12–6 L-J potential with the parameters reported by Heinz et al.[19] (r_0 = 2.616Å, ε_0 = 4.72kcal/mol), which yield realistic solid–liquid interfacial properties.

The density-derived electrostatic and chemical (DDEC) method was used to partition the electron and spin densities to compute net atomic charges (NACs) by Chargemol software[20]. The DDEC NACs are simultaneously optimized to reproduce the chemical states of atoms and the electrostatic potential, outside the material's electron distribution[21], which is well-suited for studying the chemical properties of materials and for constructing force fields used in atomistic simulations. Forcefield parameters for the electrolyte are based on the OPLS-AA force field[22]. The TIP/3P model was used for water, because of its superior ability to replicate the experimental dielectric constant of water compared to other widely used water models.[23-25] The equilibrium bond lengths, angles, and dihedrals were taken from the optimized structures performed by VASP. Sobtop[26] was used to help build the parameters that were still missing (not presented in OPLS-AA nor i.e. bond length Cu-C, angles O-C-O for CO_2 adsorption on Cu).

1.2.2 Free Energy Pertubation(FEP)

To investigate the influence of the electric double layer (EDL) on CO_2 adsorption process, we employed the Free Energy Perturbation (FEP) method to calculate the corresponding free energy changes. Each simulation window underwent energy minimization and 100 ps NVT/NPT equilibration, followed by 2 ns molecular dynamics simulations. The FEP topology was constructed with two states: the non-bonded initial state (Cu------CO_2) and the bonded final state (Cu–CO_2). In both states, the Cu–CO_2

interaction was represented by a harmonic bond potential, with the equilibrium bond length and force constant obtained from GC-DFT calculations and subsequent sob-top post-processing. Therefore, the classical MD force field itself does not explicitly include the bond formation energy of Cu–CO_2; this energetic contribution was derived from GC-DFT in an implicit solvent model. The FEP simulations were first performed in pure water environment, and the resulting free energy changes were calibrated against the GC-DFT values. Using this calibration as a reference, we then evaluated the free energy changes under various EDL environments through FEP simulations. In summary, the role of GC-DFT is twofold: to provide accurate energetic benchmarks for the reaction center and to supply structural and charge parameters for force-field parameterization, whereas the role of FEP is to capture environmental corrections to the CO_2 adsorption free energy.

We applied electric fields (EF) of −0.6 V/nm to model the EDL with electrode potentials. These values were selected based on the following considerations[27]: For the 0.1 M $KHCO_3$ electrolyte, the Debye length is ~0.96 nm at room temperature. The EF value can be calculated by applying the linearized Poisson-Boltzmann equation for the particular case of a charged plane, which is: $E = -\nabla\varphi$; $\varphi = \varphi_0 e^{-\kappa r}$. Where E is the electric field, φ is the potential, κ is the inverse of the Debye length, and *r* is the distance to the plane. Therefore, the electric field will be:

$$E = -\frac{d\varphi}{dr} = k\varphi_0 e^{-kr}$$

The mdp files used for the FEP:

```
; Run parameters
integrator              = md
nsteps                  = 2000000
dt                      = 0.001
; Output control
nstxout                 = 100000
nstvout                 = 100000
nstfout                 = 100000
nstenergy               = 200
nstlog                  = 100000
nstxout-compressed      = 200
compressed-x-grps       = System
; Bond parameters
```

```
continuation                  = yes
constraint_algorithm        = lincs
constraints                    = h-bonds
lincs_iter                      = 1
lincs_order                    = 4
; Neighborsearching
cutoff-scheme                 = Verlet
ns_type                          = grid
nstlist                         = 10
rcoulomb                        = 1.0
rvdw                              = 1.0
; Electrostatics
coulombtype                    = PME
pme_order                       = 4
fourierspacing               = 0.16
; Temperature coupling is on
tcoupl                            = V-rescale
tc-grps                          = system
tau_t                             = 0.1
ref_t                          = 300
; Pressure coupling is on
pcoupl                            = Berendsen
pcoupltype                     = semiisotropic
tau_p                             = 2.0
ref_p                            = 1.0    1.0
compressibility              = 4.5e-5    4.5e-5
refcoord_scaling             = com
;ref_p                            = 1.0
;compressibility              = 4.5e-5
; Periodic boundary conditions
pbc                                = xyz
; Dispersion correction
DispCorr                         = EnerPres
; Velocity generation
gen_vel                          = no
define                            = -DPOSRES
electric-field-z              = -0.6 0 0 0
; Free energy control parameters
free_energy                     = yes
init_lambda_state            = 0
delta_lambda                    = 0
calc_lambda_neighbors       = 1
couple-lambda0                 = vdw-q
couple-lambda1                 = vdw-q
```

couple-intramol = yes
; Vectors of lambda specified here
; init_lambda_state 0 1 2 3 4 5 6 7 8 9 10
vdw_lambdas = 0.00 0.00 0.00 0.00 0.00 0.00 0.00 0.00 0.00 0.00 0.00 0.00 0.00 0.00 0.00 0.00
coul_lambdas = 0.00 .0666 .1333 .2000 .2666 .3333 .4000 .4666 .5333 .6000 .6666 .7333 .8000 .8666 .9333 1.0000
bonded_lambdas = 0.00 .0666 .1333 .2000 .2666 .3333 .4000 .4666 .5333 .6000 .6666 .7333 .8000 .8666 .9333 1.0000
restraint_lambdas = 0.00 .0666 .1333 .2000 .2666 .3333 .4000 .4666 .5333 .6000 .6666 .7333 .8000 .8666 .9333 1.0000
; Masses are not changing (particle identities are the same at lambda = 0 and lambda = 1)
mass_lambdas = 0.00 0.00 0.00 0.00 0.00 0.00 0.00 0.00 0.00 0.00 0.00 0.00 0.00 0.00 0.00 0.00
; Not doing simulated temperting here
temperature_lambdas = 0.00 0.00 0.00 0.00 0.00 0.00 0.00 0.00 0.00 0.00 0.00 0.00 0.00 0.00 0.00 0.00
; Options for the decoupling
sc-alpha = 0.5
sc-coul = no ; linear interpolation of Coulomb (none in this case)
sc-power = 1
sc-sigma = 0.3
nstdhdl = 10
disre = simple
nstdisreout = 0

We have uploaded the GROMACS simulation trajectory files, associated topology (.top) and .xvg files containing the free energy changes from FEP calculations to the Zenodo data repository:

https://zenodo.org/records/18836283

https://zenodo.org/records/20186035

2 Result

2.1 GC-DFT calculation results

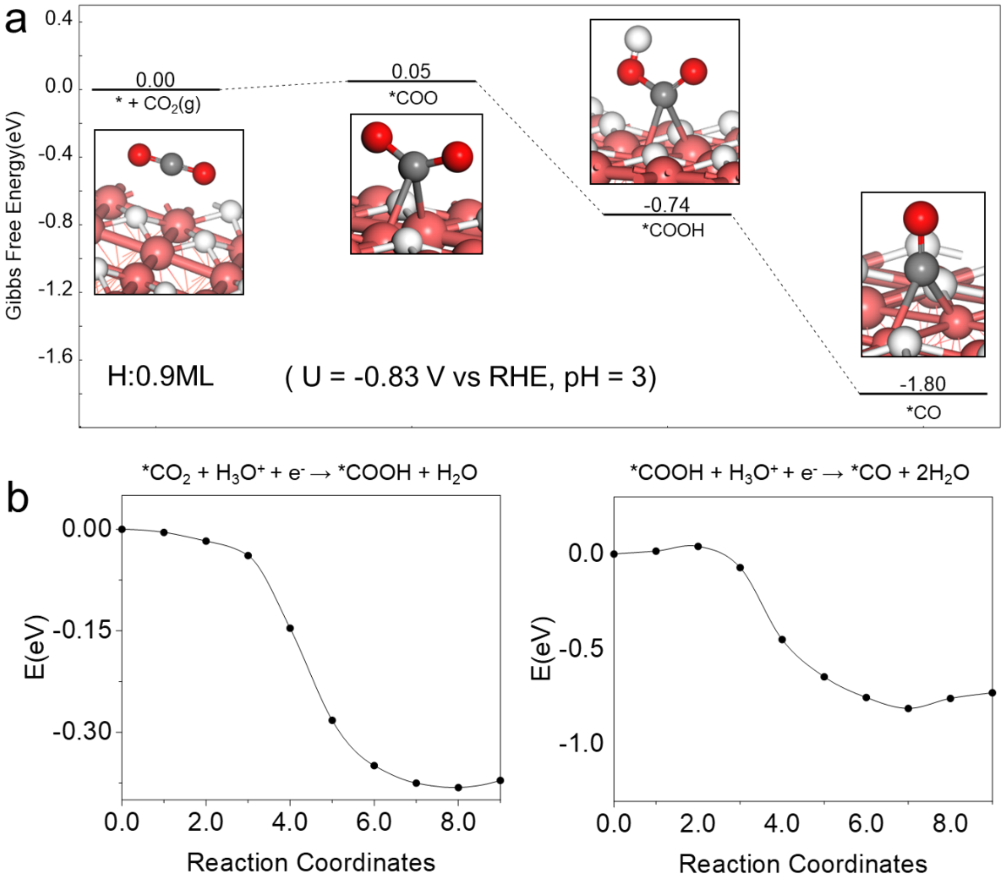

Figure S2. Reaction energetics and structural evolution during CO_2 electroreduction to *CO on a 0.9ML H coverage Cu(100) surface in acidic environment at an overpotential of –0.83 V_{RHE}. (a) Free energy profile for CO_2 reduction to *CO, with representative structures of key intermediates: *COO, *COOH, and *CO. (b) From left to right: NEB-calculated energy profiles for the first and second protonation steps.

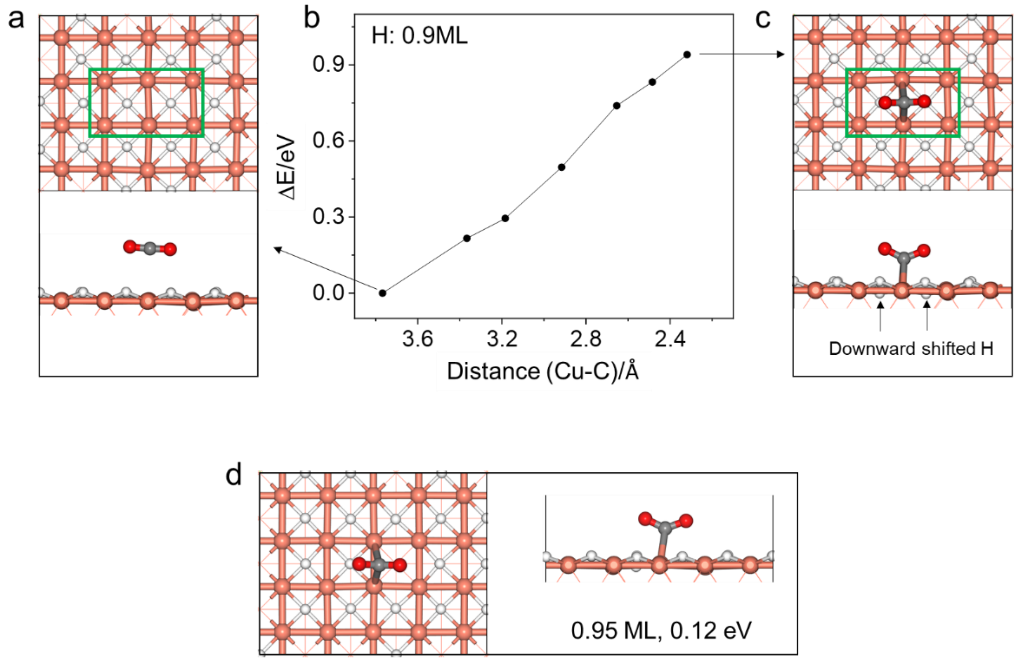

Figure S3. (a) Top and side views of CO_2 physisorption above H-occupied hollow sites (green box) on 0.9 ML H-covered Cu(100). CO_2 is omitted in the top view for clarity to show the hollow-site H atoms. (b) Energy profile for Cu–C bond-length scanning within the green box region. (c) Top and side views of CO_2 chemisorption within the green box region. (d) CO_2 adsorption configurations and corresponding adsorption energies on Cu(100) at 0.95 ML H coverage.

As shown in Figure S3a, at the 0.9 ML H-covered Cu surface, geometry optimization shows that CO_2 cannot adsorb within the region highlighted in the green box. Cu–C bond-length scanning further shows that the CO_2 adsorption barrier in this configuration(Figure S3b) is comparable to that at 1.0 ML coverage. As the Cu–C distance decreases, the hollow-site H atoms shift noticeably downward (Figure S3c). This indicates that the substantial energy difference between 0.9 and 1.0 ML H coverage shown in the main text primarily originates from the H atom at the hollow site directly beneath the adsorbed CO_2, which strongly suppresses CO_2 adsorption. We also calculated CO_2 adsorption at 0.95 ML (Figure S3d). Geometry optimization reveals that CO_2 can adsorb in a bridge configuration where one of the hollow sites directly beneath CO_2 is unoccupied by H.

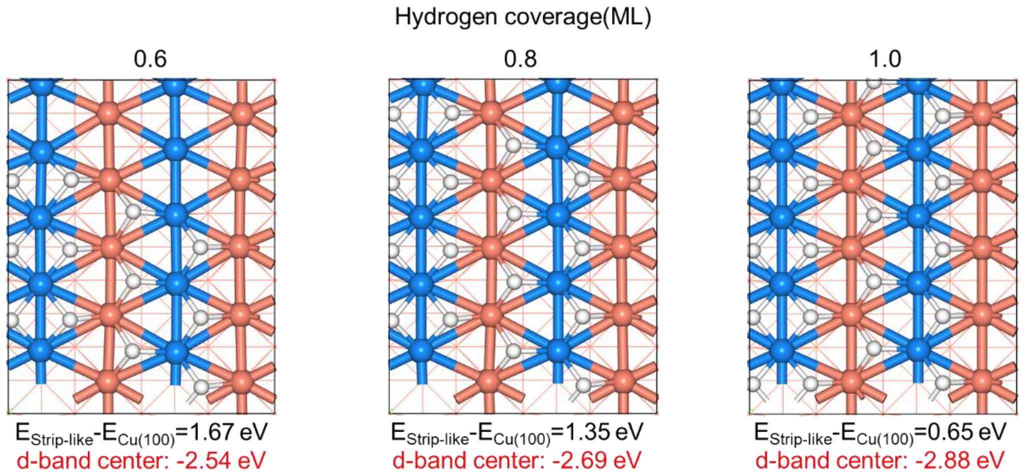

Figure S4. Stripe-like reconstructed Cu(100) with varying H coverage. $E_{Strip\text{-}like}$ - $E_{Cu(100)}$ denotes the electronic energy of the stripe-like reconstructed Cu surface relative to Cu(100) at equivalent H coverage. As H coverage increases, the stripe-like Cu surface becomes more stable. The d-band center of the surface Cu atoms shifts downward with increasing H coverage. Brick-red spheres represent original Cu atoms, where blue indicates Cu atoms in shifted rows, and white spheres represent Cu and H atoms, respectively.

Previous studies have shown that lower pH and higher overpotential generally promote reconstruction.[28-29] Magnussen and co-workers found that Cu(100) undergoes reconstruction to form stripe-like structures (shifted atomic rows as shown in Figure S4) at pH 1 to 2.75, whereas no reconstruction is observed at pH 3 or higher.[28] Given that H coverage may induce reconstruction of the Cu(100) surface, we performed constant-potential simulations at -0.83V_{RHE}. At H coverage below 0.4 ML, the stripe-like reconstructed surface reverts to Cu(100) upon geometry optimization. At H coverage between 0.6-1.0 ML, the stripe-like structure remains stable but is higher in electronic energy than Cu(100) at equivalent H coverage(Figure S4). As H coverage increases from 0.6 to 1.0ML, the stripe-like structure becomes progressively more stable relative to the H-covered Cu(100), indicating that higher H coverage thermodynamically favors stripe-like reconstruction. This implies that reconstruction of Cu(100) may occur if H coverage exceeds 1.0 ML, at -0.83 V_{RHE}. Besides, similar to Cu(100), increasing H-coverage shifts the d-band center of the strip-like structure progressively downward, suggesting that H-induced passivation of CO_2RR activity also applies to the reconstructed Cu surface.

2.2 Classical Molecular Dynamics simulations results

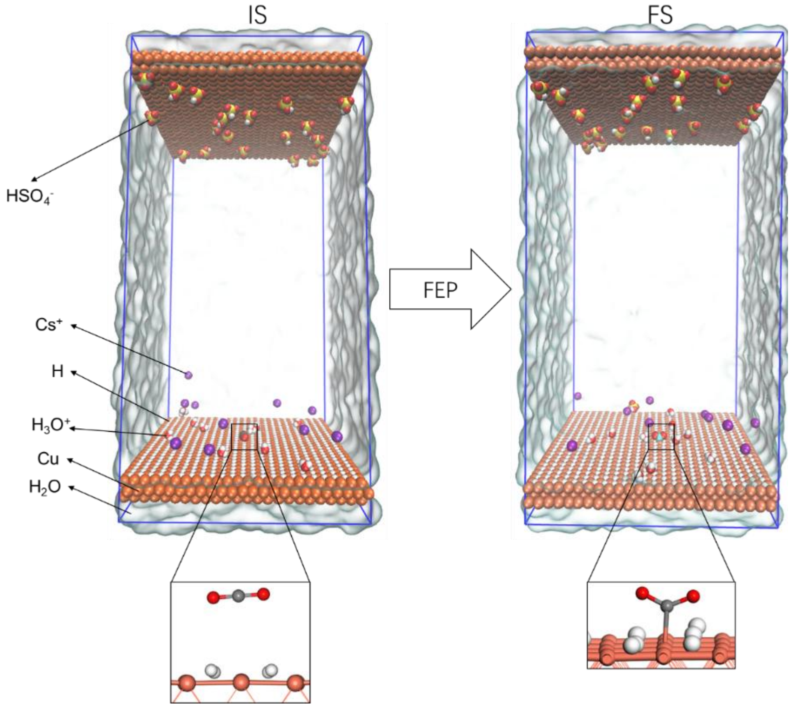

Figure S5. FEP simulations of CO_2 adsorption on Cu(100) using classical MD.

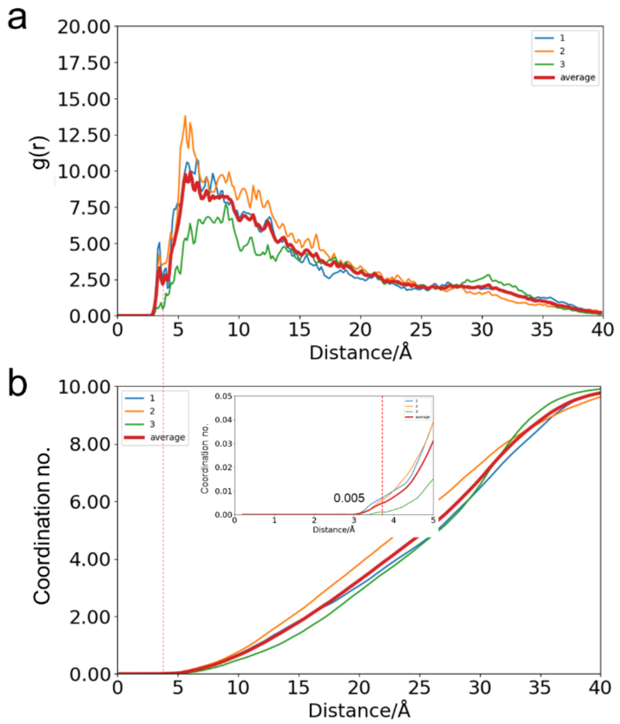

Figure S6 (a) Radial distribution function (RDF) of Cs^+ around $*COO^-$, where curves 1, 2, and 3 represent three independent MD simulations and the bold red line denotes their average. (b) The integration of the RDF.

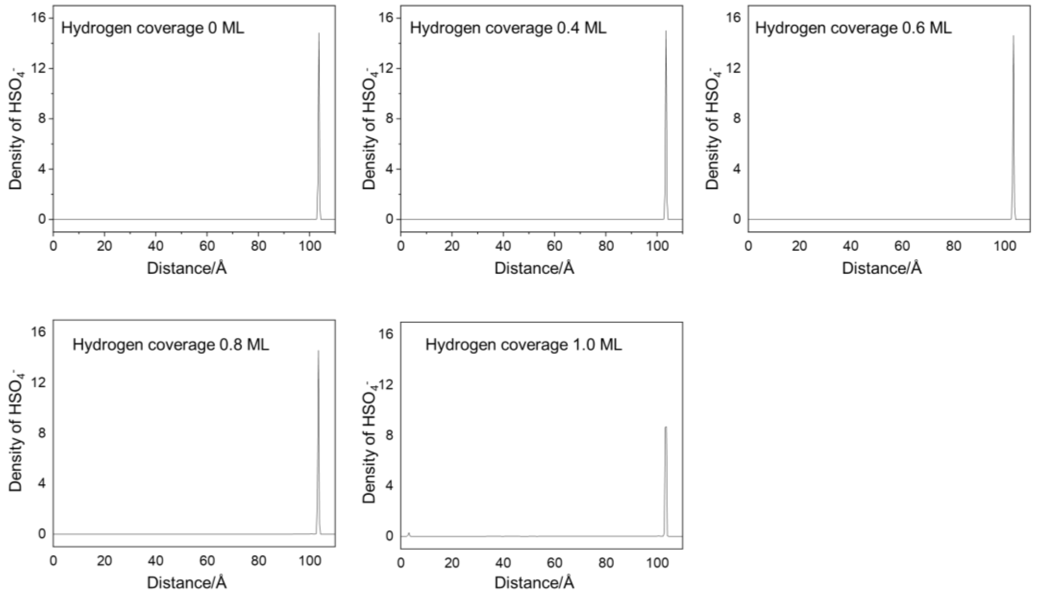

Figure S7 Distribution density of HSO_4^- ions along the z-direction above the Cu surface at varying H coverages.

3 Cartesian coordinates of optimized structures from GC-DFT calculations.

Cu(100)
1.00000000000000
10.2238998412999997 0.0000000000000000 0.0000000000000000
0.0000000000000000 12.7798995972000000 0.0000000000000000
0.0000000000000000 0.0000000000000000 38.6147003174000005
Cu
60
Direct
0.0000000000000000 0.0000000000000000 0.4509499970000022
0.1250000000000000 0.1000000009999980 0.4041399959999978
0.1250110082948055 0.1000003423072755 0.4964309995486786
0.2500000000000000 0.0000000000000000 0.4509499970000022
0.3750000000000000 0.1000000009999980 0.4041399959999978
0.3749919069495320 0.0999989978765399 0.4964295841795661
0.5000000000000000 0.0000000000000000 0.4509499970000022
0.6250000000000000 0.1000000009999980 0.4041399959999978
0.6250065505341738 0.1000001579547529 0.4964304734104346
0.7500000000000000 0.0000000000000000 0.4509499970000022
0.8750000000000000 0.1000000009999980 0.4041399959999978
0.8749904775167394 0.1000017491198051 0.4964314854189240
0.0000000000000000 0.2000000029999995 0.4509499970000022
0.1250000000000000 0.3000000119999982 0.4041399959999978
0.1250056356250226 0.3000005328133142 0.4964306012599522

0.2500000000000000	0.2000000029999995	0.4509499970000022
0.3750000000000000	0.3000000119999982	0.4041399959999978
0.3749907121447578	0.2999996694550831	0.4964316359238836
0.5000000000000000	0.2000000029999995	0.4509499970000022
0.6250000000000000	0.3000000119999982	0.4041399959999978
0.6250104705475295	0.2999994346460539	0.4964305536752320
0.7500000000000000	0.2000000029999995	0.4509499970000022
0.8750000000000000	0.3000000119999982	0.4041399959999978
0.8749920889423350	0.3000005042161860	0.4964301020646218
0.0000000000000000	0.4000000059999991	0.4509499970000022
0.1250000000000000	0.5000000000000000	0.4041399959999978
0.1250072749574116	0.4999994107887318	0.4964302880524158
0.2500000000000000	0.4000000059999991	0.4509499970000022
0.3750000000000000	0.5000000000000000	0.4041399959999978
0.3749907793274687	0.5000008659260544	0.4964310152895166
0.5000000000000000	0.4000000059999991	0.4509499970000022
0.6250000000000000	0.5000000000000000	0.4041399959999978
0.6250069532370972	0.5000000090220738	0.4964308858238056
0.7500000000000000	0.4000000059999991	0.4509499970000022
0.8750000000000000	0.5000000000000000	0.4041399959999978
0.8749940032945389	0.4999988236374691	0.4964304430024953
0.0000000000000000	0.6000000240000034	0.4509499970000022
0.1250000000000000	0.6999999880000018	0.4041399959999978
0.1250078470711955	0.6999991489911750	0.4964305411332361
0.2500000000000000	0.6000000240000034	0.4509499970000022
0.3750000000000000	0.6999999880000018	0.4041399959999978
0.3749930294479000	0.7000005809878260	0.4964303777707642
0.5000000000000000	0.6000000240000034	0.4509499970000022
0.6250000000000000	0.6999999880000018	0.4041399959999978
0.6250071634064227	0.6999998002178884	0.4964307523576323
0.7500000000000000	0.6000000240000034	0.4509499970000022
0.8750000000000000	0.6999999880000018	0.4041399959999978
0.8749922527019578	0.6999988545213185	0.4964308262792372
0.0000000000000000	0.8000000119999982	0.4509499970000022
0.1250000000000000	0.8999999759999966	0.4041399959999978
0.1250107295181948	0.9000002247345833	0.4964312096716910
0.2500000000000000	0.8000000119999982	0.4509499970000022
0.3750000000000000	0.8999999759999966	0.4041399959999978
0.3749927063105147	0.9000005282231598	0.4964304675248172
0.5000000000000000	0.8000000119999982	0.4509499970000022
0.6250000000000000	0.8999999759999966	0.4041399959999978
0.6250059561224433	0.9000003311052325	0.4964306718269924
0.7500000000000000	0.8000000119999982	0.4509499970000022
0.8750000000000000	0.8999999759999966	0.4041399959999978

0.8749905876316646 0.9000002909656928 0.4964317919400969

Cu(100) *COO(hollow)

1.00000000000000

10.2238998412999997 0.0000000000000000 0.0000000000000000

0.0000000000000000 12.7798995972000000 0.0000000000000000

0.0000000000000000 0.0000000000000000 38.6147003174000005

Cu C O

60 1 2

Direct

0.0000000000000000 0.0000000000000000 0.4509499970000022

0.1250000000000000 0.1000000009999980 0.4041399959999978

0.1249084609102624 0.0999466856789084 0.4962473833076047

0.2500000000000000 0.0000000000000000 0.4509499970000022

0.3750000000000000 0.1000000009999980 0.4041399959999978

0.3753368386123768 0.1002718605191077 0.4963559014224188

0.5000000000000000 0.0000000000000000 0.4509499970000022

0.6250000000000000 0.1000000009999980 0.4041399959999978

0.6246625011708815 0.1003646841955401 0.4963605857092901

0.7500000000000000 0.0000000000000000 0.4509499970000022

0.8750000000000000 0.1000000009999980 0.4041399959999978

0.8750753732614598 0.0999462193083858 0.4962503777403953

0.0000000000000000 0.2000000029999995 0.4509499970000022

0.1250000000000000 0.3000000119999982 0.4041399959999978

0.1247984304786058 0.3007972920547957 0.4964317449477349

0.2500000000000000 0.2000000029999995 0.4509499970000022

0.3750000000000000 0.3000000119999982 0.4041399959999978

0.3745180828347898 0.2978107187583632 0.4960475657324324

0.5000000000000000 0.2000000029999995 0.4509499970000022

0.6250000000000000 0.3000000119999982 0.4041399959999978

0.6255152561023607 0.2978394828281594 0.4960404181528588

0.7500000000000000 0.2000000029999995 0.4509499970000022

0.8750000000000000 0.3000000119999982 0.4041399959999978

0.8752570744484629 0.3007990801387450 0.4964269252706330

0.0000000000000000 0.4000000059999991 0.4509499970000022

0.1250000000000000 0.5000000000000000 0.4041399959999978

0.1253853941317189 0.4999773175569473 0.4962810494175685

0.2500000000000000 0.4000000059999991 0.4509499970000022

0.3750000000000000 0.5000000000000000 0.4041399959999978

0.3769328672888719 0.4974436870812085 0.4979138726523757

0.5000000000000000 0.4000000059999991 0.4509499970000022

0.6250000000000000 0.5000000000000000 0.4041399959999978

0.6234623135069342 0.4975905982639901 0.4978550708133582

0.7500000000000000 0.4000000059999991 0.4509499970000022

0.8750000000000000	0.5000000000000000	0.4041399959999978
0.8749167806040390	0.4999773625214701	0.4962651796254320
0.0000000000000000	0.6000000240000034	0.4509499970000022
0.1250000000000000	0.6999999880000018	0.4041399959999978
0.1227691494123064	0.6996330602990355	0.4963741516811169
0.2500000000000000	0.6000000240000034	0.4509499970000022
0.3750000000000000	0.6999999880000018	0.4041399959999978
0.3684669639540630	0.7034181909685415	0.4973244973587683
0.5000000000000000	0.6000000240000034	0.4509499970000022
0.6250000000000000	0.6999999880000018	0.4041399959999978
0.6314808974864619	0.7038415727497309	0.4973263401136023
0.7500000000000000	0.6000000240000034	0.4509499970000022
0.8750000000000000	0.6999999880000018	0.4041399959999978
0.8772409870895146	0.6996249863528377	0.4963713919221888
0.0000000000000000	0.8000000119999982	0.4509499970000022
0.1250000000000000	0.8999999759999966	0.4041399959999978
0.1250621193486054	0.8992093286805769	0.4964023300065108
0.2500000000000000	0.8000000119999982	0.4509499970000022
0.3750000000000000	0.8999999759999966	0.4041399959999978
0.3747899849535301	0.9029115548721975	0.4960493547486848
0.5000000000000000	0.8000000119999982	0.4509499970000022
0.6250000000000000	0.8999999759999966	0.4041399959999978
0.6252484569173831	0.9031235018652026	0.4960578709479151
0.7500000000000000	0.8000000119999982	0.4509499970000022
0.8750000000000000	0.8999999759999966	0.4041399959999978
0.8749679608449412	0.8991929386868520	0.4964043439232739
0.5013600649340333	0.5564013268347310	0.5377592431513420
0.5019939201999222	0.5087416404446685	0.5659907589149782
0.5000986798915150	0.6618371969557373	0.5368966094161465

Cu(100) *COO(bridge)

1.00000000000000

10.2238998412999997	0.0000000000000000	0.0000000000000000
0.0000000000000000	12.7798995972000000	0.0000000000000000
0.0000000000000000	0.0000000000000000	38.6147003174000005

Cu	C	O
60	1	2

Direct

0.0000000000000000	0.0000000000000000	0.4509499970000022
0.1250000000000000	0.1000000009999980	0.4041399959999978
0.1251652191180384	0.1002237119229861	0.4963319390024452
0.2500000000000000	0.0000000000000000	0.4509499970000022
0.3750000000000000	0.1000000009999980	0.4041399959999978
0.3752992840098770	0.0993535798972118	0.4963504341193143

0.5000000000000000	0.0000000000000000	0.4509499970000022
0.6250000000000000	0.1000000009999980	0.4041399959999978
0.6246981268443363	0.0993552671690807	0.4963503537323106
0.7500000000000000	0.0000000000000000	0.4509499970000022
0.8750000000000000	0.1000000009999980	0.4041399959999978
0.8748288801624113	0.1002251091974884	0.4963324297644718
0.0000000000000000	0.2000000029999995	0.4509499970000022
0.1250000000000000	0.3000000119999982	0.4041399959999978
0.1245426446959144	0.3004933822885363	0.4964042703622980
0.2500000000000000	0.2000000029999995	0.4509499970000022
0.3750000000000000	0.3000000119999982	0.4041399959999978
0.3737269297925181	0.2977293645837307	0.4960547866107419
0.5000000000000000	0.2000000029999995	0.4509499970000022
0.6250000000000000	0.3000000119999982	0.4041399959999978
0.6262636456459774	0.2977209368330378	0.4960550851033076
0.7500000000000000	0.2000000029999995	0.4509499970000022
0.8750000000000000	0.3000000119999982	0.4041399959999978
0.8754437382189906	0.3004919660645555	0.4964051174971402
0.0000000000000000	0.4000000059999991	0.4509499970000022
0.1250000000000000	0.5000000000000000	0.4041399959999978
0.1252555141715774	0.5000418615894731	0.4962296736290241
0.2500000000000000	0.4000000059999991	0.4509499970000022
0.3750000000000000	0.5000000000000000	0.4041399959999978
0.3767237299924346	0.4993060876571747	0.4974972605967167
0.5000000000000000	0.4000000059999991	0.4509499970000022
0.6250000000000000	0.5000000000000000	0.4041399959999978
0.6233037742995720	0.4992645752871852	0.4975092816278135
0.7500000000000000	0.4000000059999991	0.4509499970000022
0.8750000000000000	0.5000000000000000	0.4041399959999978
0.8747840852830819	0.5000344635506551	0.4962270816759897
0.0000000000000000	0.6000000240000034	0.4509499970000022
0.1250000000000000	0.6999999880000018	0.4041399959999978
0.1244116399297681	0.6996057773331543	0.4964209629313743
0.2500000000000000	0.6000000240000034	0.4509499970000022
0.3750000000000000	0.6999999880000018	0.4041399959999978
0.3734480903525181	0.7019178463112397	0.4962141462583389
0.5000000000000000	0.6000000240000034	0.4509499970000022
0.6250000000000000	0.6999999880000018	0.4041399959999978
0.6264987083467233	0.7019124685730773	0.4962242848294594
0.7500000000000000	0.6000000240000034	0.4509499970000022
0.8750000000000000	0.6999999880000018	0.4041399959999978
0.8755718816693374	0.6996094069036118	0.4964178371445058
0.0000000000000000	0.8000000119999982	0.4509499970000022
0.1250000000000000	0.8999999759999966	0.4041399959999978

0.1251908079208377	0.8997529074358397	0.4963497590562369
0.2500000000000000	0.8000000119999982	0.4509499970000022
0.3750000000000000	0.8999999759999966	0.4041399959999978
0.3752889890082187	0.9005622883558644	0.4963333648045563
0.5000000000000000	0.8000000119999982	0.4509499970000022
0.6250000000000000	0.8999999759999966	0.4041399959999978
0.6247091075763933	0.9005660893391862	0.4963338463891347
0.7500000000000000	0.8000000119999982	0.4509499970000022
0.8750000000000000	0.8999999759999966	0.4041399959999978
0.8748047337153366	0.8997550609327760	0.4963506274218901
0.5003057382580991	0.5059707642485307	0.5434838266027953
0.4999377740181785	0.4202589418380143	0.5596523030610285
0.5005979326456185	0.5982574737551047	0.5555480782703413

Cu(100) *COO(top)

1.00000000000000

10.2238998412999997	0.0000000000000000	0.0000000000000000
0.0000000000000000	12.7798995972000000	0.0000000000000000
0.0000000000000000	0.0000000000000000	38.6147003174000005

Cu	C	O
60	1	2

Direct

0.0000000000000000	0.0000000000000000	0.4509499970000022
0.1250000000000000	0.1000000009999980	0.4041399959999978
0.1250013576527778	0.1000272212005200	0.4961027795045183
0.2500000000000000	0.0000000000000000	0.4509499970000022
0.3750000000000000	0.1000000009999980	0.4041399959999978
0.3753135381917403	0.0998894190050805	0.4962692283453052
0.5000000000000000	0.0000000000000000	0.4509499970000022
0.6250000000000000	0.1000000009999980	0.4041399959999978
0.6250058113119437	0.1004425100583006	0.4963893574087592
0.7500000000000000	0.0000000000000000	0.4509499970000022
0.8750000000000000	0.1000000009999980	0.4041399959999978
0.8746510272233010	0.0998850191282870	0.4962680972705797
0.0000000000000000	0.2000000029999995	0.4509499970000022
0.1250000000000000	0.3000000119999982	0.4041399959999978
0.1250137127182214	0.3005281692225665	0.4963847890543391
0.2500000000000000	0.2000000029999995	0.4509499970000022
0.3750000000000000	0.3000000119999982	0.4041399959999978
0.3746857148591900	0.2998857246618556	0.4962142582816753
0.5000000000000000	0.2000000029999995	0.4509499970000022
0.6250000000000000	0.3000000119999982	0.4041399959999978
0.6249707426878146	0.2977662557417062	0.4960783953580403
0.7500000000000000	0.2000000029999995	0.4509499970000022

0.8750000000000000	0.3000000119999982	0.4041399959999978
0.8753128577890124	0.2998259816505069	0.4961943540954010
0.0000000000000000	0.4000000059999991	0.4509499970000022
0.1250000000000000	0.5000000000000000	0.4041399959999978
0.1249951421208877	0.5000114665003679	0.4965226888162064
0.2500000000000000	0.4000000059999991	0.4509499970000022
0.3750000000000000	0.5000000000000000	0.4041399959999978
0.3739706528126945	0.4997709037737792	0.4962248005760301
0.5000000000000000	0.4000000059999991	0.4509499970000022
0.6250000000000000	0.5000000000000000	0.4041399959999978
0.6250375280392575	0.4955645664877650	0.4988077961326525
0.7500000000000000	0.4000000059999991	0.4509499970000022
0.8750000000000000	0.5000000000000000	0.4041399959999978
0.8760076845166012	0.4997600130855275	0.4962336100592495
0.0000000000000000	0.6000000240000034	0.4509499970000022
0.1250000000000000	0.6999999880000018	0.4041399959999978
0.1250153807640828	0.6994650388052648	0.4966161623426046
0.2500000000000000	0.6000000240000034	0.4509499970000022
0.3750000000000000	0.6999999880000018	0.4041399959999978
0.3714463918038859	0.7004298964022837	0.4958354244142100
0.5000000000000000	0.6000000240000034	0.4509499970000022
0.6250000000000000	0.6999999880000018	0.4041399959999978
0.6248413681384619	0.7057187362310984	0.4980226698106875
0.7500000000000000	0.6000000240000034	0.4509499970000022
0.8750000000000000	0.6999999880000018	0.4041399959999978
0.8786058849950820	0.7004791538412221	0.4958171784050052
0.0000000000000000	0.8000000119999982	0.4509499970000022
0.1250000000000000	0.8999999759999966	0.4041399959999978
0.1250022200589740	0.8996135302762127	0.4963411685671630
0.2500000000000000	0.8000000119999982	0.4509499970000022
0.3750000000000000	0.8999999759999966	0.4041399959999978
0.3751824290872321	0.8998817525370342	0.4962428527912408
0.5000000000000000	0.8000000119999982	0.4509499970000022
0.6250000000000000	0.8999999759999966	0.4041399959999978
0.6249924218554455	0.9034508086721829	0.4961305275882708
0.7500000000000000	0.8000000119999982	0.4509499970000022
0.8750000000000000	0.8999999759999966	0.4041399959999978
0.8747856866557839	0.8999258420044143	0.4962319628707377
0.6280471719749059	0.5512277615283807	0.5468094115830340
0.6293367108735026	0.4909737341573006	0.5722125529079705
0.6276700276173202	0.6523303865131851	0.5483676333839611

Cu(100) *COOH(hollow)

1.00000000000000

```
    10.2238998412999997     0.0000000000000000     0.0000000000000000
     0.0000000000000000    12.7798995972000000     0.0000000000000000
     0.0000000000000000     0.0000000000000000    38.6147003174000005
   Cu   C    O    H
    60    1    2    1
Direct
  0.0000000000000000  0.0000000000000000  0.4509499970000022
  0.1250000000000000  0.1000000009999980  0.4041399959999978
  0.1249697584123766  0.1002555200363702  0.4963626431073180
  0.2500000000000000  0.0000000000000000  0.4509499970000022
  0.3750000000000000  0.1000000009999980  0.4041399959999978
  0.3751749249495404  0.0997560232353578  0.4963175880709443
  0.5000000000000000  0.0000000000000000  0.4509499970000022
  0.6250000000000000  0.1000000009999980  0.4041399959999978
  0.6248226291748509  0.0992511892394461  0.4963060746531340
  0.7500000000000000  0.0000000000000000  0.4509499970000022
  0.8750000000000000  0.1000000009999980  0.4041399959999978
  0.8750205696402915  0.1002397221621578  0.4963731503337030
  0.0000000000000000  0.2000000029999995  0.4509499970000022
  0.1250000000000000  0.3000000119999982  0.4041399959999978
  0.1245782207771029  0.3003021595073179  0.4965039461951051
  0.2500000000000000  0.2000000029999995  0.4509499970000022
  0.3750000000000000  0.3000000119999982  0.4041399959999978
  0.3730263596278860  0.2984997547861283  0.4958530084254917
  0.5000000000000000  0.2000000029999995  0.4509499970000022
  0.6250000000000000  0.3000000119999982  0.4041399959999978
  0.6257681867676936  0.2971665634376279  0.4962501283394189
  0.7500000000000000  0.2000000029999995  0.4509499970000022
  0.8750000000000000  0.3000000119999982  0.4041399959999978
  0.8757758324910583  0.3001045006293097  0.4962308936959090
  0.0000000000000000  0.4000000059999991  0.4509499970000022
  0.1250000000000000  0.5000000000000000  0.4041399959999978
  0.1251047938670737  0.5000785365055620  0.4964437365263095
  0.2500000000000000  0.4000000059999991  0.4509499970000022
  0.3750000000000000  0.5000000000000000  0.4041399959999978
  0.3741634656005957  0.4994547161234166  0.4965329990622394
  0.5000000000000000  0.4000000059999991  0.4509499970000022
  0.6250000000000000  0.5000000000000000  0.4041399959999978
  0.6246633439976037  0.5002540373859929  0.4989675645354055
  0.7500000000000000  0.4000000059999991  0.4509499970000022
  0.8750000000000000  0.5000000000000000  0.4041399959999978
  0.8758038755569686  0.5002103014737429  0.4962994820314819
  0.0000000000000000  0.6000000240000034  0.4509499970000022
  0.1250000000000000  0.6999999880000018  0.4041399959999978
```

0.1250643849171240	0.6997644208889753	0.4964680854920616
0.2500000000000000	0.6000000240000034	0.4509499970000022
0.3750000000000000	0.6999999880000018	0.4041399959999978
0.3751029711684524	0.7009797845909347	0.4961890015167327
0.5000000000000000	0.6000000240000034	0.4509499970000022
0.6250000000000000	0.6999999880000018	0.4041399959999978
0.6253553257905295	0.7024268049275335	0.4961511939644012
0.7500000000000000	0.6000000240000034	0.4509499970000022
0.8750000000000000	0.6999999880000018	0.4041399959999978
0.8749375097373928	0.6997310780261330	0.4964072636588597
0.0000000000000000	0.8000000119999982	0.4509499970000022
0.1250000000000000	0.8999999759999966	0.4041399959999978
0.1250162446205181	0.8998824336004532	0.4962977079988278
0.2500000000000000	0.8000000119999982	0.4509499970000022
0.3750000000000000	0.8999999759999966	0.4041399959999978
0.3752087228424514	0.8999939201763780	0.4963426798413195
0.5000000000000000	0.8000000119999982	0.4509499970000022
0.6250000000000000	0.8999999759999966	0.4041399959999978
0.6247914924293099	0.9002695468611392	0.4963657675521134
0.7500000000000000	0.8000000119999982	0.4509499970000022
0.8750000000000000	0.8999999759999966	0.4041399959999978
0.8749301927096553	0.8996948619171050	0.4963014826578629
0.5566632231592052	0.4782808181694378	0.5461871666706202
0.5623480447718663	0.3974262692291859	0.5636616003216446
0.5160646465433416	0.5673861506952420	0.5643428795706669
0.5107445876582446	0.6234862969390775	0.5464865047704492

Cu(100) *CO(hollow)
1.00000000000000

10.2238998412999997	0.0000000000000000	0.0000000000000000
0.0000000000000000	12.7798995972000000	0.0000000000000000
0.0000000000000000	0.0000000000000000	38.6147003174000005

Cu	C	O
60	1	1

Direct

0.0000000000000000	0.0000000000000000	0.4509499970000022
0.1250000000000000	0.1000000009999980	0.4041399959999978
0.1246857558170049	0.0999993505415375	0.4963032597069343
0.2500000000000000	0.0000000000000000	0.4509499970000022
0.3750000000000000	0.1000000009999980	0.4041399959999978
0.3749466499521574	0.1000102264452991	0.4960740870302587
0.5000000000000000	0.0000000000000000	0.4509499970000022
0.6250000000000000	0.1000000009999980	0.4041399959999978
0.6250529909633897	0.1000092008919253	0.4960741226353065

0.7500000000000000	0.0000000000000000	0.4509499970000022
0.8750000000000000	0.1000000009999980	0.4041399959999978
0.8753138493284141	0.0999993793722140	0.4963029942454824
0.0000000000000000	0.2000000029999995	0.4509499970000022
0.1250000000000000	0.3000000119999982	0.4041399959999978
0.1249767296553586	0.3001004462028618	0.4965305297225413
0.2500000000000000	0.2000000029999995	0.4509499970000022
0.3750000000000000	0.3000000119999982	0.4041399959999978
0.3754012511917395	0.3006788455435299	0.4963184774820562
0.5000000000000000	0.2000000029999995	0.4509499970000022
0.6250000000000000	0.3000000119999982	0.4041399959999978
0.6246013851617533	0.3006775302861797	0.4963186638224784
0.7500000000000000	0.2000000029999995	0.4509499970000022
0.8750000000000000	0.3000000119999982	0.4041399959999978
0.8750256412296906	0.3001010205204011	0.4965304672779780
0.0000000000000000	0.4000000059999991	0.4509499970000022
0.1250000000000000	0.5000000000000000	0.4041399959999978
0.1252200021360537	0.5000193263826986	0.4964102158338051
0.2500000000000000	0.4000000059999991	0.4509499970000022
0.3750000000000000	0.5000000000000000	0.4041399959999978
0.3749600521464416	0.5000266267458642	0.4981230592440511
0.5000000000000000	0.4000000059999991	0.4509499970000022
0.6250000000000000	0.5000000000000000	0.4041399959999978
0.6250503843743687	0.5000251522729542	0.4981211115320434
0.7500000000000000	0.4000000059999991	0.4509499970000022
0.8750000000000000	0.5000000000000000	0.4041399959999978
0.8747893190409215	0.5000193781843052	0.4964101013469815
0.0000000000000000	0.6000000240000034	0.4509499970000022
0.1250000000000000	0.6999999880000018	0.4041399959999978
0.1252116800020673	0.6999816545198954	0.4964094842605391
0.2500000000000000	0.6000000240000034	0.4509499970000022
0.3750000000000000	0.6999999880000018	0.4041399959999978
0.3749594679807444	0.6999775325984459	0.4981221321853653
0.5000000000000000	0.6000000240000034	0.4509499970000022
0.6250000000000000	0.6999999880000018	0.4041399959999978
0.6250501668931747	0.6999792575914228	0.4981200978335139
0.7500000000000000	0.6000000240000034	0.4509499970000022
0.8750000000000000	0.6999999880000018	0.4041399959999978
0.8747958341160569	0.6999817152137240	0.4964097021682363
0.0000000000000000	0.8000000119999982	0.4509499970000022
0.1250000000000000	0.8999999759999966	0.4041399959999978
0.1249749864207672	0.8998998292884153	0.4965299294372230
0.2500000000000000	0.8000000119999982	0.4509499970000022
0.3750000000000000	0.8999999759999966	0.4041399959999978

0.3753980094165641	0.8993360693396042	0.4963161991990219
0.5000000000000000	0.8000000119999982	0.4509499970000022
0.6250000000000000	0.8999999759999966	0.4041399959999978
0.6246052524549768	0.8993358768450150	0.4963163785568767
0.7500000000000000	0.8000000119999982	0.4509499970000022
0.8750000000000000	0.8999999759999966	0.4041399959999978
0.8750274294328122	0.8998993144168139	0.4965297690673509
0.5000143445582168	0.6000350849210179	0.5263973775243613
0.5000208266480897	0.6000667429313822	0.5581965440167238

Reference

[1] Kresse, G.; Furthmüller, J. Efficient Iterative Schemes for Ab Initio Total-Energy Calculations Using a Plane-Wave Basis Set. *Phys. Rev. B* **1996**, *54*, 11169-11186.

[2] Kresse, G.; Joubert, D. From Ultrasoft Pseudopotentials to the Projector Augmented-Wave Method. *Phys. Rev. B* **1999**, *59*, 1758-1775.

[3] Kresse, G.; Furthmüller, J. Efficiency of Ab-Initio Total Energy Calculations for Metals and Semiconductors Using a Plane-Wave Basis Set. *Comput. Mater. Sci.* **1996**, *6*, 15-50.

[4] Perdew, J. P.; Burke, K.; Ernzerhof, M. Generalized Gradient Approximation Made Simple. *Phys. Rev. Lett.* **1996**, *77*, 3865-3868.

[5] Blöchl, P. E. Projector Augmented-Wave Method. *Phys. Rev. B* **1994**, *50*, 17953-17979.

[6] Grimme, S.; Antony, J.; Ehrlich, S.; Krieg, H. A Consistent and Accurate Ab Initio Parametrization of Density Functional Dispersion Correction (DFT-D) for the 94 Elements H-Pu. *J. Chem. Phys.* **2010**, *132*, 154104.

[7] Mathew, K.; Sundararaman, R.; Letchworth-Weaver, K.; Arias, T. A.; Hennig, R. G. Implicit Solvation Model for Density-Functional Study of Nanocrystal Surfaces and Reaction Pathways. *J. Chem. Phys.* **2014**, *140*.

[8] Fernández, D. P.; Mulev, Y.; Goodwin, A. R. H.; Sengers, J. M. H. L. A Database for the Static Dielectric Constant of Water and Steam. *J. Phys. Chem. Ref. Data* **1995**, *24*, 33-70.

[9] Duan, Z.; Xiao, P. Simulation of Potential-Dependent Activation Energies in Electrocatalysis: Mechanism of O–O Bond Formation on RuO_2. *J. Phys. Chem. C* **2021**, *125*, 15243-15250.

[10] *https://github.com/penghao-xiao/Electrochemicalbarrier (accessed Mar 1, 2023.).*

[11] Peterson, A. A.; Abild-Pedersen, F.; Studt, F.; Rossmeisl, J.; Nørskov, J. K. How Copper Catalyzes the Electroreduction of Carbon Dioxide into Hydrocarbon Fuels. *Energy Environ. Sci.* **2010**, *3*, 1311-1315.

[12] Hutchison, P.; Warburton, R. E.; Soudackov, A. V.; Hammes-Schiffer, S. Multicapacitor Approach to Interfacial Proton-Coupled Electron Transfer Thermodynamics at Constant Potential. *J. Phys. Chem. C* **2021**, *125*, 21891-21901.

[13] Goodpaster, J. D.; Bell, A. T.; Head-Gordon, M. Identification of Possible Pathways for C–C Bond Formation During Electrochemical Reduction of CO_2: New Theoretical Insights from an Improved Electrochemical Model. *J. Phys. Chem. Lett.* **2016**, *7*, 1471-1477.

[14] Wilkins, D. M.; Manolopoulos, D. E.; Dang, L. X. Nuclear Quantum Effects in Water Exchange around Lithium and Fluoride Ions. *J. Chem. Phys.* **2015**, *142*, 064509.

[15] Ye, K.; Han, Y.; Wu, F.; Cheng, X.; Duan, Z.; Zhang, G.; Hu, P.; Ahlquist, M. r. S. G. How

Cation Size Modulates the Anion Effect in CO_2 Electroreduction: Insights from Multiscale Modeling of Electrochemical Interfaces. *ACS Catal.* **2025**, *15*, 17672-17677.

[16] Ye, K.; Hu, M.; Zhang, G.; Ahlquist, M. S. G. Support-Induced Interfacial Effects Steer Methanol Selectivity in CO_2 Electroreduction by Immobilized Cobalt Phthalocyanine. *Angew. Chem. Int. Ed. Engl.* **2026**, *65*, e21683.

[17] Zhu, X.; Huang, J.; Eikerling, M. Hierarchical Modeling of the Local Reaction Environment in Electrocatalysis. *Acc. Chem. Res.* **2024**, *57*, 2080-2092.

[18] Berendsen, H. J. C.; van der Spoel, D.; van Drunen, R. Gromacs: A Message-Passing Parallel Molecular Dynamics Implementation. *Comput. Phys. Commun.* **1995**, *91*, 43-56.

[19] Heinz, H.; Vaia, R. A.; Farmer, B. L.; Naik, R. R. Accurate Simulation of Surfaces and Interfaces of Face-Centered Cubic Metals Using 12-6 and 9-6 Lennard-Jones Potentials. *J. Phys. Chem. C* **2008**, *112*, 17281-17290.

[20] Manz, T. A.; Limas, N. G. Introducing Ddec6 Atomic Population Analysis: Part 1. Charge Partitioning Theory and Methodology. *RSC Adv.* **2016**, *6*, 47771-47801.

[21] Liu, S.; Luan, B. Benchmarking Various Types of Partial Atomic Charges for Classical All-Atom Simulations of Metal-Organic Frameworks. *Nanoscale* **2022**, *14*, 9466-9473.

[22] Jorgensen, W. L.; Maxwell, D. S.; Tirado-Rives, J. Development and Testing of the Opls All-Atom Force Field on Conformational Energetics and Properties of Organic Liquids. *J. Am. Chem. Soc.* **1996**, *118*, 11225-11236.

[23] Jorgensen, W. L. Quantum and Statistical Mechanical Studies of Liquids. 10. Transferable Intermolecular Potential Functions for Water, Alcohols, and Ethers. Application to Liquid Water. *Chem. Inform.* **2002**, *12*.

[24] Ryckaert, J.-P.; Ciccotti, G.; Berendsen, H. J. C. Numerical Integration of the Cartesian Equations of Motion of a System with Constraints: Molecular Dynamics of N-Alkanes. *J. Comput. Phys.* **1977**, *23*, 327-341.

[25] Alexiadis, A.; Kassinos, S. Molecular Simulation of Water in Carbon Nanotubes. *Chem. Rev.* **2008**, *108*, 5014-5034.

[26] Tian Lu, S., Version [Version 1.0], http://sobereva.com/soft/Sobtop (accessed on Dec. 26, 2023).

[27] Stojek, Z. In *Electroanalytical Methods: Guide to Experiments and Applications*, Scholz, F., Bond, A. M., Compton, R. G., Fiedler, D. A., Inzelt, G., Kahlert, H., Komorsky-LovriÄ‡, Å., Lohse, H., LovriÄ‡, M., Marken, F., Neudeck, A., Retter, U., Scholz, F., Stojek, Z., Eds. Springer Berlin Heidelberg: Berlin, Heidelberg, 2010; pp 3-9.

[28] Matsushima, H.; Haak, C.; Taranovskyy, A.; Grunder, Y.; Magnussen, O. M. In Situ Video Stm Studies of the Hydrogen-Induced Reconstruction of Cu(100): Potential and Ph Dependence. *Phys. Chem. Chem. Phys.* **2010**, *12*, 13992-8.

[29] Zhang, Z.; Wei, Z.; Sautet, P.; Alexandrova, A. N. Hydrogen-Induced Restructuring of a Cu(100) Electrode in Electroreduction Conditions. *J. Am. Chem. Soc.* **2022**, *144*, 19284-19293.
